# Development of New Cancer Treatment by Identifying and Focusing the Genetic Mutations or Altered Expression in Gynecologic Cancers

**DOI:** 10.3390/genes12101593

**Published:** 2021-10-09

**Authors:** Yun-Hsin Tang, Chiao-Yun Lin, Chyong-Huey Lai

**Affiliations:** 1 Department of Obstetrics and Gynecology, Chang Gung Memorial Hospital Linkou Branch and Chang Gung University College of Medicine, Taoyuan 333, Taiwan; 8902037@cgmh.org.tw (Y.-H.T.); chiao.yun0101@gmail.com (C.-Y.L.); 2 Gynecologic Cancer Research Center, Chang Gung Memorial Hospital Linkou Branch and Chang Gung University, Taoyuan 333, Taiwan

**Keywords:** homologous recombination repair, mismatch repair, ovarian cancer, endometrial cancer, gynecologic cancer

## Abstract

With the advent of next-generation sequencing (NGS), The Cancer Genome Atlas (TCGA) research network has given gynecologic cancers molecular classifications, which impacts clinical practice more and more. New cancer treatments that identify and target pathogenic abnormalities of genes have been in rapid development. The most prominent progress in gynecologic cancers is the clinical efficacy of poly(ADP-ribose) polymerase (PARP) inhibitors, which have shown breakthrough benefits in reducing hazard ratios (HRs) (HRs between 0.2 and 0.4) of progression or death from *BRCA1/2* mutated ovarian cancer. Immune checkpoint inhibition is also promising in cancers that harbor mismatch repair deficiency (dMMR)/microsatellite instability (MSI). In this review, we focus on the druggable genetic alterations in gynecologic cancers by summarizing literature findings and completed and ongoing clinical trials.

## 1. Introduction

According to the Global Cancer Statistics 2020, cervical cancer, corpus cancer, and ovarian cancer were the fourth, sixth, and seventh in incidence and fourth, eleventh, and seventh in mortality rates among female malignancies [1]. Epithelial ovarian cancer (EOC) accounts for 90% of ovarian malignancies. Approximately 75% of EOC patients are diagnosed at an advanced stage with a 5-year survival rate of around 20-30% [2]. Uterine corpus cancer, mainly diagnosed in early stages, is associated with favorable survival, except for advanced stage or aggressive histologic types [3]. Cancer of the uterine cervix is mainly caused by viral etiology (human papillomavirus (HPV) infections), while different integration signatures related to HPV genotypes were found [4]. Aggressive chemotherapy, radiotherapy, and targeted therapy with anti-angiogenesis agents have improved survival of gynecologic cancers [5,6,7]. However, patients with recurrent/refractory diseases have rapid progression, and most of them will die of disease. There are still unmet needs in current treatment in gynecologic cancers.

**Figure 1:**
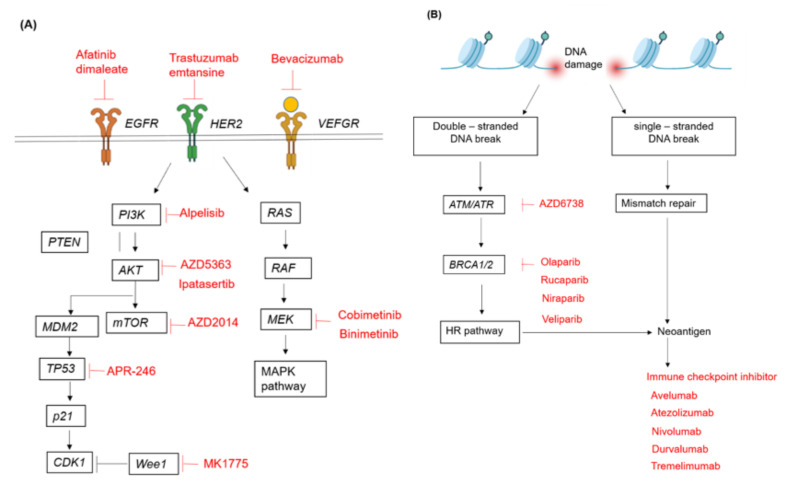
Altered pathways in gynecologic cancers. (**A**) The regulatory functions of oncogenic and tumor suppressor genes in the RTK (receptor tyrosine kinase) signaling pathway. (**B**) DNA damage repair pathways. The inhibitors against gene describe the rationale of therapies in cancer treatment (signed by red words).

With the advent of next-generation sequencing (NGS), The Cancer Genome Atlas (TCGA) research network has given gynecologic cancers molecular classifications, which impacts clinical practice more and more [8,9,10]. EOC has more frequent mutations in *TP53, FOXM1, RB, PI3K/RAS, NOTCH* pathway, and homologous recombination (HR) alterations. Endometrial cancer has more frequent mutations in the *PI3K/AKT* and *RTK/RAS/β-catenin* pathway. Cervical cancers exhibited genomic alterations in either one or both of the PI3K-MAPK and *TGFβ* pathways, illustrating the potential clinical significance of therapeutic agents targeting members of these pathways [8,9,10] (Figure 1).

New cancer treatments that identify and target pathogenic abnormalities of genes have been in rapid development. In this review, we focus on the druggable genetic alterations in gynecologic cancers by summarizing literature findings and the clinical efficacy of clinical trials. We further describe the ongoing trials and potential drugs that are under development.

## 2. Alterations in Homologous Recombination Pathway as a Biomarker and Target of Cancer Therapies

### 2.1. BRCA1/2 Mutations

The homologous recombination (HR) pathway plays a pivotal role in the repair of DNA double-strand breaks (DSBs) and inter-strand crosslinks, which maintain genomic stability by cooperation with the Faconi Anemia (FA) pathway [8]. Approximately 50% of epithelial ovarian cancers (EOC) harbor genetic and epigenetic alterations of the HR pathway genes [8]. Women with diagnosed EOC should have germline or somatic genetic testing for *BRCA1/2* variants [11]. Germline *BRCA1* and *BRCA2* mutations are the most common genetic alterations, which are observed in 15 to 20% of all EOCs [2]. Ovarian cancer patients with germline *BRCA* mutations had a better survival rate with a generally favorable response to platinum-based chemotherapy, compared to patients who were *BRCA*-wild type [8,12,13,14,15]. Somatic mutation of *BRCA1* and *BRCA2* have been identified in 6% of EOCs [8]. A total of 81% of *BRCA1* and 72% of *BRCA2* mutations are heterozygous loss, and the majority of germline and somatic mutations are frameshift insertions or deletions [8]. 

### 2.2. Homologous Recombination Repair Genes beyond BRCA1/2

In addition to *BRCA1/2*, the homologous recombination repair (HRR) genes that have been identified and applied in studies are ATM, *ATR, BARD1, BLM, BRIP1, CDK12, CHEK1, CHEK2, FANCA, FANCC, FANCD2, FANCE, FANCF, FANCI, FANCL, FANCM, MRE11, NBN, PALB2, RAD50, RAD51, RAD51B, RAD51C, RAD51D, RAD52, RAD54L, and RPA1* [16,17,18]. The homologous recombination deficiency (HRD) score integrates three independent DNA-based measurements of genomic instability, including loss of heterozygosity (LOH), telomeric allelic imbalance (TAI), and large-scale transitions (LST) in the tumor tissue [19]. HRD has been shown to be a predictive biomarker of PARPi therapy beyond BRCA status [19]. 

### 2.3. Poly(ADP-ribose) Polymerase (PARP) Inhibition in Epithelial Ovarian Cancer

#### 2.3.1. *PARP* and PARP Inhibitor

PARP inhibitors (PARPi), the first synthetic lethal drugs that are the first clinically approved, are targeting in HRD cancers, which have the defect in the homologous recombination repair pathway, the conservative mechanism of repair of DSBs [20]. PARP enzymes involve a number of cellular pathways that regulate energy metabolism, gene transcription, cell death, and epigenetic modifications [21,22,23]. There are 17 members in the *PARP* family, and *PARP1, PARP2,* and *PARP3* are related to DNA repair [24]. *PARP* shares a synthetic lethal relationship with *BRAC1/2*, both of which are key in DNA double-strand break repair [24,25]. With PARP inhibition, persistent single-strand DNA breaks (SSBs), which are repaired through active base-excision repair pathways, lead to the accumulation of double-strand breaks. In HRD cancer cells, those DSBs are not repaired, which leads to cell death. Other mechanisms of PARP inhibitors include PARP1 trapping, activation of error-prone nonhomologous end joining (NHEJ), and impaired BRCA1 recruitment [20,24,26,27,28,29,30,31].

PARPis, including olaparib (AZD2281, KuDOS/AstraZeneca), niraparib (MK4827, Merck/Tesaro), rucaparib (CO338, AG014699, and PF01367338, Pfizer/Clovis), and veliparib (ABT888, Abbvie), all interact with the binding site of the PARP enzyme cofactor, β-NAD+, in the catalytic domain of *PARP1* and *PARP2.* PARPi have been extensively studied in epithelial ovarian cancers (Table 1) [32,33,34,35,36,37,38,39,40,41,42]. A second-generation PARPi, talazoparib (Lead/Biomarin/Medivation/Pfizer), has been developed. Talazoparib is more potent in trapping PARP1 protein on DNA, preventing autoPARylation and *PARP1* release from the site of damage, interfering with the catalytic cycle of PARP1, and has a higher in-vitro cytotoxicity in *BRCA* mutant cells compared with olaparib [20,43]. Talazoparib has been approved for adults with deleterious or suspected deleterious germline *BRCA*-mutated, human epidermal growth factor receptor 2 (*HER2*)-negative, locally advanced, or metastatic breast cancer, but with limited evidence in EOCs [20,44,45].

#### 2.3.2. Clinical Trials of PARP Inhibitors in Epithelial Ovarian Cancer

##### Olaparib

Study 19, a phase II randomized, double-blinded trial, evaluated maintenance therapy with olaparib in recurrent platinum-sensitive high-grade serous ovarian cancer patients who received two or more lines of platinum-based chemotherapy and had a partial or complete response in their most recent platinum-based regimen. In this trial, PFS was longer in patients that received olaparib 400 mg twice daily (8.4 vs. 4.8 months; HR, 0.35; *p* < 0.001) [32].

In Study 42, a phase II single arm trial, an overall response rate of 34% was observed in 137 advanced EOC patients who had received three or more lines of chemotherapy and who had a measurable disease at baseline with germline *BRCA1/2* mutation (g*BRCA1/2*m) with oral 400 mg olaparib twice daily. The median progression-free survival was 6.7 months [33]. Based on this study, the US Food and Drug Administration (FDA) approved olaparib as a treatment for patients with g*BRCA*m ovarian cancer who had received three or more lines of chemotherapies in 2014 [34]. 

SOLO-1, an international, randomized, double-blinded, phase III trial, evaluated the efficacy of olaparib as a maintenance therapy in newly diagnosed, advanced, high-grade serous or endometrioid ovarian cancer, primary peritoneal cancer, or tubal cancer with germline or somatic *BRCA* mutations (*BRCA*m). In this study, the risk of disease progression or death was reduced at 70% with a hazard ratio (HR) of 0.30 (*p* < 0.001) [35]. In 2018, the US FDA extended the indication of olaparib monotherapy for first-line maintenance treatment in *BRCA*m advanced ovarian cancer [46]. 

In the PAOLA-1 trial, patients with newly diagnosed, advanced, high-grade ovarian cancer, having a response after first-line platinum-taxane chemotherapy plus bevacizumab were included, regardless of their BRCA status. In this phase III, randomized, double-blinded trial, the median progression-free survival (PFS) was 22.1 months with olaparib plus bevacizumab as a first-line maintenance, and it was 16.6 months with a placebo plus bevacizumab (HR 0.59, *p* < 0.001). The treatment benefit was seen in both HRD-positive/*BRCA*m tumors (HR 0.33) and HRD-positive/*BRCA*-wild type tumors (HR 0.43) [36]. Based on this result, the US FDA further expanded the approval of olaparib to include its use in combination with bevacizumab for first-line maintenance treatment in HRD-positive advanced ovarian cancer [46].

##### Rucaparib

In addition to olaparib, several other *PARP* inhibitors have had promising results in phase II and phase III trials. ARIEL2, a phase II, open-label trial, evaluated rucaparib in patients with recurrent platinum-sensitive, high-grade, serous or endometrioid ovarian cancers. PFS was significantly longer in the *BRCA* mutant (12.8 vs. 5.2 months; HR, 0.27; *p* < 0.0001) and LOH high (5.7 vs. 5.2 months; HR, 0.62; *p* = 0.011) subgroups compared with the LOH low subgroup [37].

In the ARIEL3 trial, a phase III, randomized, double-blinded trial in recurrent platinum-sensitive high-grade serous or endometrioid ovarian cancers, rucaparib showed significant treatment benefits compared with the placebo in all three biomarker groups that were defined based on the NGS assay that included *BRCA*-mutant (*BRCA*m) (PFS 16.6 months vs. 5.4 months, HR 0.23, *p* < 0.0001), *BRCA* wild type (*BRCA*wt)/loss of heterozygosity (LOH)-high (PFS 9.7 months vs. 5.4 months, HR 0.44, *p* < 0.0001), and *BRCA*wt/LOH-low (PFS 6.7 months vs. 5.4 months, HR 0.58, *p* = 0.0049) [38,39]. Rucaparib has been granted by the FDA for the maintenance of recurrent EOCs in a complete or partial response to platinum-based chemotherapy in 2018. 

##### Niraparib

The efficacy in ovarian cancer treatment of niraparib, a potent *PARP*i, was mainly evaluated by two randomized, double-blinded, phase III trials, the ENGOT-OV16/NOVA trial and the PRIMA trial, which lead to the FDA approvals of niraparib for the maintenance treatment in patients with recurrent, platinum-sensitive EOCs. It was also approved for those with newly diagnosed advanced epithelial ovarian, fallopian tube, or primary peritoneal cancer who are in a complete or partial response to first-line, platinum-based chemotherapy. In the ENGOT-OV16/NOVA trial, 553 platinum-sensitive, recurrent EOC patients were enrolled and randomly assigned to receive niraparib 300 mg or a placebo once daily. In this trial, patients who received niraparib had a significantly longer median PFS in all three subgroups: g*BRCA*m cohort (21 months vs. 5.5 months, HR 0.27, *p* < 0.001), HRD/g*BRCA*wt cohort (12.9 months vs. 3.8 months, HR 0.38, *p* < 0.001), and HR-proficient (HRP)/g*BRCA*wt cohort (9.3 months vs. 3.9 months, HR 0.45, *p* < 0.001) [40]. 

The PRIMA study randomized 733 newly diagnosed advanced EOC patients who had complete or partial response to first-line, platinum-based chemotherapy to receive niraparib 300 mg or a placebo once daily as maintenance therapy. Patients in the niraparib group had a significantly longer median PFS in not only the HRD category (21.9 months vs. 10.4 months, HR 0.43, *p* < 0.001), but also in overall population (13.8 months vs. 8.2 months, HR 0.62, *p* < 0.001) [41]. 

The most common grade three or four adverse events (AEs) of *PARP*is are anemia (17–31%), fatigue (1.9–9%), nausea/vomiting (1–8%), thrombocytopenia, and neutropenia, and they were predominantly found in NOVA and PRIMA trials (28.7–33.8% and 12.8–19.6%, respectively) [33,35,36,38,39,40,41,47,48]. 

In addition to *PARP*i monotherapy, there are increasing interests in combination therapy with *PARP* inhibitors, including an immune checkpoint inhibitor, anti-*VEGF mTOR* inhibitors, and select trials that are summarized in Table 2.

### 2.4. PARP Inhibitors in Endometrial Cancer 

According to The Cancer Genome Atlas data, the HRD phenotype was also recently reported in 25% of uterine endometrial carcinomas [49]. It occurs largely restricted to non-endometrioid, *TP53*-mutated endometrial cancers and represents nearly 50% of the cases [50]. In TP53 wild-type endometrioid carcinoma, the most common molecular alterations, *PTEN* or *ARID1A*, have been associated with significant in vitro *PARP* inhibitor activity [51,52]. Those characteristics increased the interest of *PARP*i in uterine cancers. There is an ongoing randomized phase II trial, UTOLA trial, to evaluate olaparib as maintenance therapy in platinum-sensitive advanced uterine cancer. Other ongoing trials are selected and summarized in Table 2.

## 3. Mismatch Repair (MMR)/Microsatellite Instability (MSI)

### 3.1. MMR Deficiency (dMMR) and MSI in Gynecologic Cancer

DNA mismatch repair (MMR) plays a key role in genomic stability by identifying and repairing base-base mismatches and insertion/deletion mismatches during DNA replication and recombination [53]. Microsatellite sequences are segments of repeated DNA (usually 10–60 base pair) composed of several base pairs (usually 1–6 base pair) that repeat sequentially [54,55]. These DNA segments are susceptible to mutations since they are prone to DNA polymerase pausing and slippage during DNA replication due to their repetitive nature [54,56]. MMR, as a part of the DNA repair system, maintains the repeat count of microsatellites during cell division by recognizing the newly replicated DNA and repairing the DNA mutations [53,57]. Mismatch repair deficiency (dMMR) leads to cells being unable to regulate the lengths of their microsatellites, which results in microsatellite instability (MSI) and malignancies, including gynecologic cancers [55,58]. 

MMR deficiencies are known to occur through inherited germline MMR pathway mutations or somatic mutations. Lynch syndrome, an inherited disease with the mutation of the MMR genes (*MLH1, MSH2, MSH6,* or *PMS2*) or deletion of the stop codon of the EPCAM genes, is one of the most prevalent hereditary cancer-prone syndromes. Besides colorectal cancer, Lynch syndrome is associated with increased frequencies of cancers of the endometrium, stomach, small bowel, hepatobiliary system, upper urologic tract, and ovary [59,60]. In germline dMMR population, the cumulative endometrial cancer risk at 70 years is highest in *MLH1* mutations (34–54%), followed by *MSH2* (21–51%), *MSH6* (16–49%), and *PMS2* (13–24%) mutations. The cumulative ovarian cancer is highest in *MSH2* mutations (15%), followed by *MLH1* mutations (11%) [58,61]. Epigenetic alterations, such as hypermethylation of the *MLH1* promoter, epigenetic inactivation of *MSH2*, or downregulation of MMR genes by miRNAs, can suppress transcription and interfere with the expression of MMR genes and cause dMMR [58,62,63,64]. Hypermethylation of the MLH1 promoter is the most common cause of sporadic dMMR/MSI [58]. 

dMMR was found in over 17–33% of endometrial cancers, 3.5% of uterine carcinosarcomas, and in 2.6% of cervical squamous cell carcinomas and endocervical adenocarcinomas [55]. dMMR is found in approximately 10–12% of epithelial ovarian cancers with a predominance in endometrioid (19.2%), mucinous (16.9%), and clear cell (11.5%) histology, whereas the incidence of dMMR in serous cancers has been reported to be 1–8% [65,66,67]. The most frequent mutations were *MSH2* (47%) and *MLH1* (38%) in women with Lynch syndrome and who were diagnosed with epithelial ovarian cancer with a favorable 10-year overall survival [68]. Undifferentiated and dedifferentiated endometrial carcinoma, an undifferentiated carcinoma mixed with differentiated endometrioid carcinoma, was also reported with approximately 50% of MMR deficiency [69]. 

### 3.2. Detection of MSI

There are several clinically available MSI detection methods, including NGS with the accuracy of 92–94.6%, Fluorescent multiplex PCR and CE (gold standard) with the accuracy of 100%, immunochemistry (IHC) stain of MMR proteins with the accuracy of 89–95%, and smMIPs with the accuracy of 95.8% [70,71,72,73,74]. High microsatellite instability (MSI-H), low microsatellite instability (MSI-L), and microsatellite stability (MSS) are classified according to the frequency of MSI. MSI-H is historically defined as instability in two or more of the five markers in the Bethesda reference panel (BAT-25, BAT-26, D2S123, D5S346 and D17S250) or, as detected by PCR, whereas instability in only one marker is considered to be MSI-L [75,76]. In more expanded microsatellite panels, MSI-H is defined as instability in more than 30–40% of the markers and MSI-L as alteration in 10–30% of the markers [76,77]. In colorectal cancers, the concordance between PCR and IHC was as high as 96%, but a much lower concordance of 68% was reported in ovarian cancer [58]. Thus, the detection methods and panels in gynecologic cancers need to be further evaluated. 

### 3.3. Targeting Dmmr/Msi-High Gynecologic Cancer

Mismatched repair-deficient tumors have high MSI and harbor 10–100 times more mutations that encode potential neoantigens than MMR-proficient tumors [78]. Expression of programmed death ligand 1 (PD-L1), a tumor immune checkpoint, on the cell membrane of dMMR tumors has been reported [79]. Furthermore, increased CD8+ tumor-infiltrating lymphocytes (TILs) and overexpression of programmed death 1 (*PD-1*) on the TILs and peritumoral lymphocytes have been also found [79]. Increased CD8+ TILs, higher CD8+/CD4+ ratio, and higher PD-1 positive TILs were found in ovarian clear cell carcinoma with MSI, which may have benefitted from immunotherapies [80]. Those immunogenic signatures in dMMR tumors render them susceptible to immune checkpoint blockades that reactivate T cells for an antitumor response. 

Anti-PD-1/PD-L1 immunotherapy has been shown to be effective in a wide range of cancers, including ovarian cancers, cervical cancer, and endometrial cancer. Pembrolizumab (Keytruda, Merck & Co., NJ, USA.), a humanized immunoglobulin G4 monoclonal antibody that blockades *PD-1* on lymphocytes that allow the reactivation of T cell-mediated tumor killing, received accelerated approval by the US FDA in 2017 for the treatment of adult and pediatric patients with unresectable or metastatic MSI-H/dMMR solid tumors that have progressed after prior standard treatment and have no satisfactory alternative treatment options [81,82]. 

In KEYNOTE-158, a nonrandomized, open-label, multisite phase II trial, pembrolizumab (200 mg every 3 weeks, for 35 cycles) showed an objective response rate (ORR) of 57.1% with a median PFS of 25.7 months in 49 MSI-H/dMMR endometrial cancer patients (8 with complete response (CR) and 20 with partial response (PR)) and the ORR was 33.3% with a median PFS of 2.3 months in 15 MSI-H/dMMR ovarian cancer patients (three with CR and two with PR) [83]. 

In the KEYNOTE-146/Study 111 trial, pembrolizumab was combined with Lenvatinib (Lenvima, Eisai, Tokyo, Japan), an oral multikinase inhibitor that targets vascular endothelial growth factor receptors (VEGF) 1–3, fibroblast growth factor receptors (FGFR) 1–4, platelet-derived growth factor (PDGF) -α, RET, and KIT for the treatment of previously treated endometrial cancer patients. A total of 108 patients were enrolled in this phase Ib/II study. In 11 patients with dMMR/MSI-H tumors, the ORR was 63.6%, whereas the ORR was 36.2% in MMR-proficient (pMMR)/MSS tumors [84], which led to an accelerated approval by the US FDA of this combination to be used in pMMR endometrial cancer.

## 4. Tumor Suppressor Gene *TP53*

### 4.1. Tumor Suppressor p53 in Gynecologic Cancers

Tumor suppressor gene *TP53* is the most frequently mutated gene in high-grade serous adenocarcinoma of the ovary and the endometrium, which is found in 96% of ovarian high-grade serous carcinoma and in more than 90% of endometrial serous adenocarcinoma [8,9]. Tumor suppressor *p53* functions as a major barrier to neoplastic transformation. As the principal cellular responder to stress signals, including oncogene activation, DNA damage, and hypoxia, *p53* induces cell cycle arrest to keep genomic stability or apoptosis, senescence, or ferroptosis to eliminate abnormal or unrecoverable cells [85,86]. The majority of *TP53* mutations are missense mutations that produce a single amino acid substitution in the protein’s DNA-binding domain. Mutant *p53* may interact with many transcription factors such as *p63, p73, NF-kB, ATM*, and *SMADs*, altering the transcription, cell cycle, apoptosis, and metabolism of cancer cells, resulting in oncogenic gain-of-function. These changes lead to genetic instability, proliferation, metastasis, and chemoresistance. The missense mutations are divided into two categories: DNA contact mutations such as R248Q and R273H, and conformational mutations such as R249S, G245S, R175H, and R282W. These six hotspots account for nearly one third of all *p53* mutations and may be considered as targets for cancer treatment.

### 4.2. Treatment Strategies in Cancer Harboring TP53 Mutation

#### 4.2.1. Small-Molecule-Based Therapy Targeting Mutant p53

APR-246 (PRIMA-1MET), a methylated analogue of PRIMA-1, is a prodrug that is converted to the active compound methylene quinuclidinone that binds to cysteine residues in mutant p53 and restores its wild-type function [86,87]. APR-246 showed synergistic effects with cisplatin, carboplatin, doxorubicin, or gemcitabine, in ovarian cancer cell lines and re-sensitized platinum-resistant ovarian cancer cells [87]. There are several phase I/II trials of APR-246 in combination with carboplatin or doxorubicin in high grade serous ovarian cancer, and the results are pending (Table 3).

Adavosertib (MK-1775), a potent, small-molecule WEE1 kinase inhibitor, showed an antitumor effect with the combination of chemotherapy and radiotherapy in preclinical studies [88,89]. Since p53 is mainly responsible for the G1-S cell cycle arrest, p53 mutant cancer cells are more dependent on G2-M checkpoints to maintain genomic stability in the presence of DNA damage. *WEE1* is a tyrosine kinase that is involved in DNA damage induced G2-M cell cycle arrest by regulating CDK1 activity. Inhibition of WEE1, combined with DNA-damaging agents, causes the inactivation of the G2-S checkpoint, leading to unscheduled mitotic entry of cells without completion of DNA repair and replication, and it results in mitotic catastrophe and cell death in p53 mutant-harboring tumor cells [86,90]. In a phase II randomized, double-blinded trial, a total of 121 patients with TP53-mutated, platinum-sensitive recurrent ovarian cancer were randomized to an oral adavosertib 225mg twice daily for 2.5 days every 21 days or a placebo plus carboplatin and paclitaxel. In this phase II trial, the median progression-free survivals were 7.9 and 7.3 months for the adavosertib group and the placebo group, respectively, HR, 0.63; *p* = 0.08) [90]. In another phase II randomized, double-blinded trial, patients with recurrent platinum-resistant or platinum-refractory high-grade serous ovarian cancer were treated with gemcitabine and adavosertib 175 mg or an identical placebo once daily on days 1, 2, 8, 9, 15, and 16, in 28-day cycles under disease progression [91]. Whole-exome sequencing showed 95% and 100% positive of TP53 mutation in the adavosertib group and the placebo group, respectively. In this trial, patients who received adavosertib plus gemcitabine had longer PFS compared to patients with placebo plus gemcitabine (4.6 vs. 3.0 months; HR, 0.55; *p* = 0.015) and a longer median OS (11.4 vs. 7.2 months; HR, 0.56; *p* = 0.017). The most frequent grade three or more adverse events were neutropenia, anemia, and thrombocytopenia in these two trials [90,91]. A phase II trial of adavosertib in recurrent uterine serous carcinoma also showed substantial activity (ORR 29.4%, median PFS 6.1 months) [92]. There are several ongoing trials of adavosertib in combination with systemic chemotherapy or PARPi in gynecologic cancers, and they are listed in Table 3.

#### 4.2.2. Adoptive Cellular Therapy Targeting p53 Neoantigens

Adoptive cell therapy is a personalized immunotherapy that transplants autologous or allogeneic immune cells, including tumor-infiltrating lymphocytes (TIL), and immune cells with or without genetic modifications, for cancer treatment. Autologous TIL has shown a durable response in patients with solid tumors, including melanoma, breast, and colon cancers. TILs generate adaptive immune response based on recognition of unique tumor neoantigen through immunogenic T-cell receptors. TILs from resected metastatic ovarian cancers that recognized two TP53 mutation hotspots, Y220C and G245S, were identified in a recent study [93]. Beyond TIL, after in vitro stimulation with p53 neoantigens, the selected and expanded CD4+ and CD8+ antigen experienced memory T cells from peripheral blood lymphocytes of patients with a mutated TP53 tumor, which also showed T-cell responses to the mutant p53 [94]. These preclinical studies showed a new treatment strategy targeting TP53 mutation and possibilities of clinical benefits in cancers with TP53 mutations, such as high grade serous ovarian cancer.

## 5. Genetic Alterations Associated with Virus Infection

### 5.1. HPV as an Initiating Agent for Cervical Carcinogenesis

There is strong epidemiological and molecular biological evidence indicating that HPV plays a crucial role in the etiology of cervical cancer [95]. The HPV oncoproteins, E6 and E7, inhibit p53 and pRb, respectively, causing alterations of DNA repair, apoptosis, and angiogenesis, which eventually result in carcinogenesis [96,97]. High-risk HPV types also induce mitotic defects and genomic instability and cause specific mutation signatures, primarily the apolipoprotein B mRNA editing catalytic polypeptide-like (APOBEC) mutation [98,99]. 

### 5.2. HPV Integration Site 

Recent studies have reported that the most frequent integration sites of HPV were in the *MACROD2, MIPOL1/TTC6, TP63, ERBB2, KLF12, and RAD51B* gene by next-generation sequencing (NGS) in 272 Cervical cancer patients from the BioRAIDs study [NCT02428842] [4]. HPV integration sites that are within or in close proximity to several fragile sites in the *MYC, ERBB2, TP63, FANCC, RAD51B, and CEACAM5* may trigger genome instability and the nearest copy number amplification as well as increased expression of adjacent genes [100]. 

### 5.3. Copy Number Alterations in HPV-Related Cervical Carcinoma

HPV integration events affect all chromosomes, including some previously described at 3q26.31 (*TERC, MECOM*), 3q28 (*TP63*), 8q24.21 (*MYC, PVT1*), 11q22.1 (*YAP1, BIRC2, BIRC3*), and 17q12 (*ERBB2*) in cervical cancer and recurrent focal amplification events have been identified at 7p11.2 (*EGFR*), 9p24.1 (*CD274, PDCD1LG2*), 13q22.1 (*KLF5*), and 16p13.13 (*BCAR4*). In addition to previously identified deletions, at 4q35.2 (*FAT1*) and 10q23.31 (*PTEN*) and recurrent deletions were identified at 3p24.1 (*TGFBR2*) and 18q21.2 (*SMAD4*). Among those, *ERBB2, CD274 (PD-L1)*, and *PDCD1LG2 (PD-L2)* had amplifications that highlight the potential for clinical trials of ERBB2 inhibitors and immunotherapeutic strategies for a subset of cervical cancers [10,101].

## 6. Other Druggable Targets Associated with Genetic Alterations in Gynecologic Cancers

### 6.1. PI3K/AKT/mTOR Pathway

Somatic loss of phosphatase and tensin homolog (PTEN) is one of the most common genomic aberrations in endometrioid endometrial cancer, which were found in 43–46% of cases [9,102,103]. Homozygous PTEN deletion, caused by focal deletion at 10q23.31, has been found in 7% of high-grade serous ovarian cancers and is associated with downregulation of PTEN at the mRNA level [8]. In cervical cancer, aberrations in PIK3CA also tended to co-occur with PTEN somatic mutations, suggesting potential therapeutic benefits from PI3K-pathway-targeting agents [9].

PTEN alterations in cervical cancer are around 8% and are mostly due to missense and nonsense mutations [103]. As a tumor suppressor, PTEN inhibits the activation of the cell’s pro-survival signaling pathway, phosphoinositide 3-kinase (PI3K)/AKT pathway, which is important in initiation and progression of endometrial cancer [104,105]. PTEN plays a fundamental role in the maintenance of chromosomal stability through the physical interaction with centromeres and control of DNA repair, and it regulates the expression of RAD51, a key protein of the HR pathway [106]. Inhibition of PI3K in PTEN mutated cells has been shown to reduced RAD51 levels and sensitize these cells to PARPi [107]. 

Moreover, cases of EMSY amplification and PTEN homozygous deletions, which may cause HRD, and the CCNE1 amplifications, which are associated with HR proficiency, were identified [26]. PTEN deficiency is also thought to be associated with transcriptional downregulation of RAD51, which may have the potential to be treated with PARP inhibition, though some studies showed that PTEN and RAD51 are independent [108]. Mutation of RTK/RAS-PI3K pathway was found to be related to the resistance of BETi treatment in cancers, including ovarian cancers [109].

Monotherapy with PI3K/AKT/mTOR inhibitors in gynecologic cancers, however, has been shown to have a limited clinical benefit, and no drug is approved by the US FDA in gynecologic cancer currently [110]. Since activation of AKT is shown to be related to PARPi resistance in recent studies, there are some ongoing trials trying to combine PI3K/AKT/mTOR inhibitors and PARPi in gynecologic cancers [111] (Table 2). 

### 6.2. ARID1A

BRG1-associated factor (BAF) is an important tumor suppressor and the most frequently disrupted subunit of *ARID1A* [112]. This is a component of the BAF/PBAF complex, which involves transcriptional effects in polycomb silencing, DNA accessibility for transcription, and splicing patterns, as well as DNA repair and maintenance of chromatin topology and 3D architecture [113]. 

ARID1A have since been observed at high frequency in a number of studies, including uterine and ovarian clear cell carcinoma (46–57%), ovarian endometrioid carcinoma (30%), and uterine endometrioid carcinoma (47–60%) [113,114,115,116]. Some studies showed that MMR deficiency is associated with the loss of ARID1A expression in ovarian clear cell carcinoma [117]. BRD inhibition showed promising anticancer effects in some preclinical studies in clear cell carcinoma models [118]. However, phase I studies of BRD inhibitors showed dose-limited toxicities, including nausea, thrombocytopenia, and extended fatigue, which brought the obstacles for clinical use [119]. Studies showed that the loss of *ARID1A* increased microsatellite instability through deficient recruitment of *MMR* genes, which enhanced mutational burden and sensitized tumors to PD-L1 blockade [112,120].

In ovarian cancer, mutations of *ARID1A* are frequently found with activating mutations of *PI3K* [113]. Activating mutations of *PI3K* may lead to altered BAF localization or function. In the mouse model, ovarian tumors with similar features to ovarian clear cell carcinoma were only developed when with *ARID1A/PI3K* double mutations, but not with only a single *ARID1A* or *PI3K* mutation, suggesting the effects the cooperation of these two genes have in cancer [121]. Recent reports suggest new approaches for targeting tumors with altered *BAF/PBAF* complexes based on synthetic lethality. For example, tumors with *ARID1A* mutations often depend on *ARID1B*. Targeting these genetic dependencies represents a novel strategy to attack these tumors. 

Inhibition of BRD4 may cause a synergy effect with PARPi, which makes it a therapeutic target for tumors that harbor ARID1A alterations [122]. Cyclin-E1 (CCNE1) gene amplification is presented in 15% of ovarian cancers [8]. Cyclin-E1 (CCNE1) is found as a potential therapeutic target for *ARID1A*-mutated ovarian clear cell carcinoma through synthetic lethality [116]. *CCNE1* gain and *RB1* loss discriminate patients with tumors extremely sensitive to platinum retreatment [123,124]. There are some ongoing trials that target *ARID1A*-associated cancers (Table 3).

### 6.3. Potential Targets in DNA Damage Repair and Synthetic Lethality beyond PARP Inhibitors

The encouraging results of PARPis led to an increasing interest in cancer research focused on targeting various pathways involved in DNA damage repair by synthetic lethal approaches. The ataxia telangiectasia and Rad3-related (ATR) inhibitors, DNA-dependent protein kinase (DNA-PK) inhibitors, WEE1 inhibitors, and checkpoint kinase 1/2 (CHK1/2) inhibitors have shown promising clinical results recently, and a number of ongoing trials are focusing on gynecologic cancers [25,125] (Table 2 and Table 3). On the other hand, mutations of *CDK12* were found in 3% of EOCs. As one of the nine significantly mutated genes in ovarian cancer, *CDK12* involves the transcription of BRCA1 and other DNA repair genes. Disabling of *CDK12* shows reduced *BRCA1* levels, impaired HR repair, and increased sensitivity to the PARP inhibitor, and combination therapy with CDK12 inhibitor and PARPi is a potential treatment to overcome resistance of PARPi [126]. 

### 6.4. Other Druggable Targets That Are under Development

A prospective phase II trial revealed that trastuzumab in combination with paclitaxel and carboplatin significantly prolonged PFS more than chemotherapy alone in 41 stage III, IV HER2/neu over-expressed serous endometrial cancer patients (17.9 months vs. 9.3 months, HR 0.40, *p* = 0.013) [127]. However, there is no phase III study ongoing after the initial success, probably because of the rarity of such a tumor. Only a biomarker-driven study involving trastuzumab entansine is ongoing (Table 3). the enhancer of zeste 2 (EZH2) is a key epigenetic regulator of gene expression and is frequently overexpressed in cancers, including ovarian cancer and endometrial cancer [128,129]. MAPK1 mutations and the known role of the MAPK signaling pathway in cancer suggest the possibility that the mutant MAPK1 may exert oncogenic activity in cervical cancer [130]. Therapeutic agents for CCNE1, EZH2, MAPK1, and the other identified variants, including the FGFR family, MYC, MET, KRAS, and cell cycle checkpoints, are currently under investigation in active and ongoing clinical trials (Table 3). 

## 7. Conclusions

The most prominent progress in gynecologic cancers is the clinical efficacy of PARPi; their remarkable benefits in reducing HRs of progression or death from BRCA 1/2m ovarian cancer is breath-taking. Immune checkpoint inhibition in combination with targeted therapy is also promising. New cancer treatments that identify and target pathogenic abnormalities of genes will make many breakthroughs in the years to come.

## Figures and Tables

**Table 1:** Results of phase II/III clinical trials of PARP inhibitors in ovarian cancer.

Study/NCT Identifier	Design	Patient No.	Treatment	Patient Population	Efficacy	AE ≥ Grade 3	Genetic Testing
**Olaparib** (Lynparza, AstraZeneca)							
Study 19 [32], NCT00753545	Phase II, double-blind, randomized	265	Olaparib 400 mg vs. placebo orally, BID (1:1)	Recurrent platinum-sensitive, HGSOC, ≥ 2 platinum-based chemotherapy	PFS: 8.4 mo vs. 4.8 mo; HR, 0.35; *p* < 0.001 *BRCA^mut^*: 11.2 vs. 4.3 mo, HR 0.18, *p* < 0.0001 *BRCA^wt^*: 7.4 vs. 5.5 mo, HR 0.54, *p* = 0.0075 ORR: 12% vs. 4% (*p* = 0.12)	Fatigue (6.6%) Anemia (5.1%) Nausea/vomiting (4.4%) Diarrhea (2.2%)	
Study 42 [33], NCT01078662	Phase II, single arm	154	Olaparib 400 mg orally, BID	Recurrent or progressive EOCs, g*BRCA*^mut^*,* ≥ 3 lines of chemotherapy	PFS: 6.7 mo Platinum sensitive: 9.4 mo Platinum-resistant: 5.5 mo ORR: 34% (*n* = 137 with measurable disease)	Fatigue (7%) Anemia (20%) Nausea/vomiting (4%) Myelodysplatic syndrome and/or AML: 2%	Myriad’s BRACAnalysis CDx
SOLO 1 [35], NCT01844986	Phase III, double-blind, RCT	391	Olaparib 300 mg vs. placebo orally, BID (2:1) Maintenance up to 24 mo	Newly diagnosed, advanced, HGSOC or endometrioid OC, g*BRCA*1/2^mut^, CR or PR after platinum-based chemotherapy	70% risk reduction for disease progression or death PFS: 56 vs. 13.8 mo; HR 0.33, *p* < 0.001 PFS at 5 years: 48% vs. 21%	Fatigue (4%) Anemia (22%) Nausea/vomiting (1.1%) Neutropenia (9%) Thrombocytopenia (1%)	Myriad’s BRACAnalysis CDx (germline) Foundation One CDx (tissue)
PAOLA-1/ENGOT-ov25 [36], NCT02477644	Phase III, double-blind, RCT	806	Olaparib 300 mg orally BID (24 mo) + bevacizumab (15 mo) 15 mg/kg every 3 weeks vs. placebo + bevacizumab (15 mo) (2:1) Maintenance	Newly diagnosed, advanced high-grade EOC, CR or PR after platinum-taxane based chemotherapy	67% risk reduction for disease progression or death PFS: All: 22.1 vs. 16.6 mo; HR 0.56, *p* < 0.001 BRCAmut: 37.2 vs. 21.7 mo, HR 0.31 HRD: 37.2 vs. 17.1 mo, HR 0.33 HRD/BRCAwt: 28.1 vs. 16.6 mo, HR 0.43 BRCAmut: HR 0.31 HRP: 16.9 vs. 16.0 mo, HR 0.92	Fatigue (5%) Anemia (17%) Nausea/vomiting (3%) Neutropenia (6%) Thrombocytopenia (2%) Hypertension (19%)	Myriad’s myChoice^®^ HRD CDx assay (HRD: tumor score ≥ 42)
**Rucaparib** (Rubraca, Clovis)							
ARIEL2 [37] (NCT01891344)	Phase III, part 1	204	Rucaparib 600 mg orally, BID, 28 day cycles	Recurrent platinum-sensitive HGOC	PFS: BRCAmut: 12.8 mo, HR 0.27, *p* < 0.0001 LOHhi: 5.7 mo, HR 0.62, *p* = 0.011 LOHlo: 5.2 mo	Fatigue (9%) Anemia (22%), Nausea/vomiting (6%) Neutropenia (7%) Thrombocytopenia (2%)	Foundation Medicine’s T5 next-generation sequencing assay for tumor HRD and genomic LOH (LOHhi: genomic LOH ≥ 14%) Methylation-sensitive PCR for BRCA1 and RAD51C promoter hypermethylation
ARIEL3 [38,39] (NCT01968213)	Phase III, RCT	564	Rucaparib 600 mg orally BID vs. placebo (2:1)	Recurrent HGSOC or endometrioid OC with response to the last platinum-based chemotherapy	PFS: BRCAmut: 16.6 vs. 5.4 mo, HR 0.23, *p* < 0.0001 HRD: 13.6 vs. 5.4 mo, HR 0.32, *p* < 0.0001 BRCAwt/LOHhi: 9.7 vs. 5.4 mo, HR 0.44, *p* < 0.0001 BRCAwt/LOHlo: 6.7 vs. 5.4 mo, HR 0.58, *p* = 0.0049	Fatigue (7%) Anemia (22%) Nausea/vomiting (8%) Neutropenia (8%) Thrombocytopenia (5%) Increased ALT or AST (10%) 2 treatment-related deaths	Myriad’s BRCAnalysis CDx test (germline) Foundation Medicine’s T5 next-generation sequencing assay (tissue)
**Niraparib** (Zejula, Tesaro)							
ENGOT-OV16/NOVA [40] (NCT01847274)	Phase III, RCT, double-blind	533	Niraparib 300 mg orally QD vs. placebo (2:1) maintenance therapy	Recurrent platinum-sensitive, EOCs (HGSOC predominant), ≥ 2 platinum-based chemotherapy	PFS: gBRCAmut: 21.0 vs. 5.5 mo, HR 0.27, *p* < 0.001 HRD/gBRCAwt: 12.9 vs. 3.8 mo, HR 0.38, *p* < 0.001 HRP/gBRCAwt: 9.3 vs. 3.9 mo, HR 0.42, *p* < 0.001	Fatigue (8.2%) Anemia (25.3%) Nausea/vomiting (4.9%) Neutropenia (19.6%) Thrombocytopenia (33.8%) Hypertension (8.2%)	Myriad’s BRACAnalysis CDx (germline) Myriad’s myChoice^®^ HRD CDx assay (tissue) (HRD: tumor score ≥ 42)
PRIMA/ENGOT-OV26/GOG-3012 [41] (NCT02655016)	Phase III, RCT, double-blind	733	Niraparib 300 mg orally QD vs. placebo (2:1) as maintenance therapy	Newly diagnosed, advanced EOCs (HGSOC predominant), CR or PR after first-line platinum-based chemotherapy	PFS: HRD: 21.9 vs. 10.4 mo, HR: 0.43, *p* < 0.001 HRP: 8.1 vs. 5.4 mo, HR 0.68 All population: 13.8 vs. 8.2 mo, HR 0.62, *p* < 0.001	Fatigue (1.9%) Anemia (31%) Nausea/vomiting (2%) Neutropenia (12.8%) Thrombocytopenia (28.7%)	myChoice^®^ HRD CDx assay (tissue) (HRD: tumor score ≥ 42)
**Veliparib** (ABT-888, AbbVie)							
VELIA [42] (NCT02470585)	Phase III, RCT	1140	Carboplatin/paclitaxel plus Veliparib 150 mg orally BID then Veliparib 400 mg BID as maintenance Veliparib 150 mg orally BID then placebo as maintenance Placebo followed by placebo as maintenance (1:1:1)	Newly diagnosed advanced HGSOC	PFS: benefit only in Veliparib maintenance group All: 23.5 vs. 17.3 mo, HR 0.68, *p* < 0.001 BRCAmut: 34.7 vs. 22.0 mo, HR 0.44 HRD: 31.9 vs. 20.5 mo, HR 0.57, *p* < 0.001 HRD/BRCAwt: HR, 0.74 HRP/BRCAwt: HR, 0.81	Fatigue (8%) Anemia (38%) Nausea/vomiting (12%) Neutropenia (58%) Thrombocytopenia (28%)	Myriad BRACAnalysis CDx (germline)or myChoice HRD CDx assay (tissue) (HRD: tumor score ≥ 33)

**Table 2:** Select ongoing trials or trials with results pending of PARP inhibitor studies in gynecologic cancers.

ClinicalTrials.gov (Accessed Date 6 September 2021) Identifier/Study	Design	Target	Site of Cancer	Drug	Estimated Participants	Population
NCT02855944/ARIEL4	Phase III	PARP	OV	Rucaparib vs. Chemotherapy	345	Recurrent *BRCA^mut^* HGSOC
NCT04734665/NIRVANA-R	Phase II	PARP/VEGF	OV	Niraparib, Bevacizumab	44	Platinum-sensitive recurrent EOCs
NCT03326193	Phase II	PARP/VEGF	OV	Niraparib, Bevacizumab	105	Advanced EOCs post first-line platinum-based chemotherapy with bevacizumab
NCT03278717/ICON9	Phase III	PARP/VEGF	OV	Olaparib, Cediranib	618	Platinum-sensitive recurrent EOCs
NCT03642132/JAVELIN OVARIAN PARP 100	Phase III	PARP/PD-L1	OV	Talazoparib, Avelumab	79	Advanced EOCs
NCT03598270/ANITA	Phase III, RTC	PARP/PD-L1	OV	Niraparib, Atezolizumab	414	Recurrent platinum-sensitive EOCs
NCT03522246/ATHENA	Phase III, RTC	PARP/PD-1	OV	Rucaparib Nivolumab	1000	Advanced EOCs with response to first-line platinum-based chemotherapy
NCT02953457	Phase I/II	PARP/PD-L1/CTLA-4	OV	Olaparib Durvalumab Tremelimumab	40	Recurrent *BRCA1/2^mut^* EOCs
NCT03737643/DUO-O	Phase III, RTC	PARP/PD-L1/VEGF	OV	Olaparib, Durvalumab, Bevacizumab Carboplatin+Paclitaxel	1374	Newly diagnosed advanced EOCs
NCT04669002	Phase IIa/b	PARP/topoisomerase-1	OV	Olaparib, EP0057	60	Advanced EOCs with or without previous PARPi
NCT03462342/CARPI	Open-label	PARP/ATR	OV	Olaparib, AZD6738	86	Recurrent EOCs
NCT04729387/EPIK-O	Phase III	PARP/PI3K	OV	Olaparib, Alpelisib	358	Platinum resistant HGSOCs, *BRCA^wt^*
NCT02208375	Phase Ib	PARP/mTORC/AKT	OV, EM	Olaparib, AZD2014, AZD5363	159	Recurrent EM ca and EOCs
NCT03651206/ROCSA	Phase II/III	PARP/PD-1	OV, EM	Niraparib, TSR-042	196	Recurrent EM or OV carcinosarcoma
NCT04716686	Open-label	PARP	EM	Niraparib	83	Recurrent EM serous carcinoma
NCT03660826	Phase II	PARP/PD-L1/VEGF	EM	Olaparib, Cediranib, Durvalumab, Capivasertib	120	Recurrent/persistent/metastatic endometrial cancer
NCT03951415/DOMEC	Phase II	PARP/PD-L1	EM	Olaparib, Durvalumab	55	Advanced/recurrent/refractory/metastatic EMCA, including carcinosarcoma
NCT03694262/EndoBARR	Phase II	PARP/PD-1/VEGF	EM	Rucaparib, Atezolizumab, Bevacizumab	30	Recurrent or progressive EMCA

**Table 3:** Ongoing trials of targeted therapy in *ARID1A* and other genetic alterations in gynecologic cancers.

ClinicalTrials.gov (Accessed Date 6 September 2021) Identifier/Study	Design	Associated Genetic Alteration	Target	Drug	Estimated Participants	Population
NCT04104776	Phase I/II	*ARID1A^mut^*	EZH2	CPI-0209	268	OCCC/EMCA, *ARID1A^mut^*
NCT05023655	Phase II	*ARID1A^mut^*	EZH2	Tazemetostat	40	Solid tumors, *ARID1A^mut^*
NCT04493619	Phase Ib/IIa	*ARID1A^mut^*	BRD4	PLX2853 +/− Carboplatin	67	PLX2853 monotherapy: *ARID1A^mut^* advanced gynecologic cancers PLX2853+carboplatin: platinum-resistant EOC
NCT02059265	Phase II	BAF250a*^mut^*	SFK	Dasatinib	35	Recurrent or persistent EOCs and endometrial clear cell carcinoma
NCT02730923/VICTORIA	Phase I/II	PTEN*^mut^*	mTORC1/mTORC2	AZD2014 Anastrozole	72	Metastatic hormone receptor-positive EM adenocarcinoma
NCT04931342	Phase II	AKT*^mut^* BRAF/MEK*^mut^* HER2 amplification or mutations	AKT BRAF/MEK HER2	Ipatasertib Cobimetinib Trastuzumab entansine Atezolizumab + Bevacizumab	200	Persistent or recurrent epithelial ovarian cancer, fallopian tube, or primary peritoneal tumors.
NCT04729387	Phase III	No BRCA*^mut^*	PARP/PIK3CA	Alpelisib+olaparib vs Paclitaxel or Pegylated liposomal doxorubicin (PLD)	326	Platinum resistant or refractory high-grade serous ovarian cancer, with no germline BRCA mutation
NCT03345784	Phase I	p53	WEE1	Adavosertib (MK-1775) + radiotherapy + cisplatin	33	Cervical cancer, vaginal cancer, uterine cancer
NCT02098343	Phase Ib/II	p53	p53	Carboplatin with or without APR-246	200	Recurrent platinum-sensitive high-grade serous ovarian cancer
NCT02465060	Phase II	MATCH screening	Wee1, EGFR MAPK BRAF/MEK AKT PI3K NTRK HER2 PIK3CA HER2 ALK PD1 CDK4/6 ERK Hedgehog	Adavosertib; Afatinib dimaleate; Binimetinib; Capivasertib; Copanlisib; Crizotinib; Dabrafenib; Dasatinib; Defactinib; Erdafitinib; Ipatasertib; Larotrectinib; Nivolumab; Osimertinib; Palbociclib; Pertuzumab GSK2636771B; Relatlimab Sapanisertib; Sunitinib Malate Taselisib; Trametinib Trastuzumab; Trastuzumab Emtansine; Ulixertinib; Vismodegib	6420	Advanced refractory solid tumors (including ovarian, cervical cancer and corpus cancer), Lymphomas, or Multiple Myeloma

## Abbreviations

AE	Adverse event
BID	Twice daily
CR	Complete response
DSB	Double-strand break
EM	Endometrium
EMCA	Endometrial cancer
EOC	Epithelial ovarian cancer
HGOC	High-grade ovarian cancer
HGSOC	High-grade serous ovarian cancer
HR	Hazard ratio
HRD	Homologous-recombination deficiency
HRP	Homologous-recombination proficiency
LOH	Loss of heterozygosity
NGS	Next-generation sequencing
OCCC	Ovarian clear cell carcinoma
ORR	Overall response rate
PARP	Poly(ADP-ribose) polymerase
PD-L1	Programmed death ligand 1
PFS	Progression-free survival
PR	Partial response
RCT	Randomized control trial
TIL	Tumor-infiltrating lymphocyte
